# Association between *ACTN3* R577x and the physical performance of Chinese 13 to 15-year-old elite and sub-elite football players at different positions

**DOI:** 10.3389/fgene.2023.1038075

**Published:** 2023-03-10

**Authors:** Shidong Yang, Wentao Lin, Mengmeng Jia, Haichun Chen

**Affiliations:** ^1^ Department of Physical Education, Nanjing Xiaozhuang University, Najing, Jiangsu, China; ^2^ School of Physical Education and Sport Science, Fujian Normal University, Fuzhou, Fujian, China; ^3^ Department of Physical Education, Zhuhai University of Science and Technology, Zhuhai, Guangdong, China

**Keywords:** A-actinin-3, players’ positions, elite youth football players, physical performance, genotype and allele frequencies

## Abstract

The purpose of this study was to investigate the prevalence of *ACTN3* polymorphisms in Chinese elite and sub-elite football players aged 13–15 years at different positions. Specifically we explored whether *ACTN3* genotypes were linked with athletic performance of elite and sub-elite players at different positions. The RR genotype frequency of elite defenders (*p* = 0.018) and midfielders (*p* = 0.008) was significantly higher than that of sub-elite XX genotype in elite players. Furthermore, the R allele frequency of elite defenders (*p* = 0.003) and midfielders (*p* = 0.008) was significantly higher than that of sub-elite players. In all subjects, RR players performed faster and exhibited more explosive power than RX or XX players. RR, RX and XX elite players’ 20 m/30 m sprint, 5 × 25-m repeated sprint ability (5 × 25 m RSA), and standing long jump were stronger than sub-elite players, but there was no significant different in aerobic endurance between elite and sub-elite players at different positions. In conclusion, there were significant differences in *ACTN3* genotypes and alleles between elite and sub-elite players at different positions, and the RR genotype was significantly associated with power-related athletic performance in Chinese youth football players.

## Introduction

Over the past 20 years, the study of gene polymorphisms and performance in elite athletes has developed rapidly, and several genes that can be used for the identification of talented athletes have been discovered. However, previous studies on the relationship between gene polymorphisms and performance in athletes have mainly focused on the athletes whose main characteristics were strength, speed or aerobic endurance. In recent years, increasing numbers of researchers have paid specific attention to the study of the relationship between performance and gene polymorphisms in team sports (e.g., football), which collectively requires strength, speed and endurance. However, there are few studies on the genotypes of football players at different field position, especially for youth football players.

Alpha-actinin (ACTN) is a binding protein of actinin. There are two forms of actinin (α-actinin 2 and α-actinin 3) in human skeletal muscles, which are distributed in the Z line of skeletal muscles, and whose function is to anchor actinin filaments in a structural manner (Moran et al., 2007; Malyarchuk et al., 2018). The *ACTN3* gene R577X polymorphism (rs 1815739, also called the C1747 polymorphism) is a C/T polymorphism that exists in exon 16 (North et al., 1999; Suminaga et al., 2000; Vincent et al., 2010), which causes the codon encoding the amino acid at position 577 to change from CGA (encoding arginine R) to TGA (termination signal, non-coding protein), resulting in the loss of α-actinin 3. Yang and his co-workers suggested that *ACTN*3 was required for optimal fast-muscle fiber performance in strength and speed athletes, and that *ACTN3* was also beneficial for endurance athletes (Yang et al., 2003).

The role of other factors in regulating muscle fiber types has not been determined (Delmonico et al., 2007). Vincent et al. (2007) reported that the proportion and relative area of type IIx fibers in RR subjects were significantly higher than those in XX subjects. Many studies have reported that power/speed athletes (e.g., 100 m sprint, rowing, or speed skating) have a higher frequency of the RR genotype and an R allele distribution compared to the general population or endurance athletes (Eynon et al., 2009; Ahmetov et al., 2011; Cięszczyk et al., 2011; Ma et al., 2013). Moreover, endurance athletes were found to have a higher frequency of the XX genotype and an X allele distribution compared to the general population or power/speed athletes (Shang et al., 2010; Grealy et al., 2013; Pimenta et al., 2013). However, some studies have suggested that the *ACTN3* was not associated with endurance athletes (Döring et al., 2010; Grealy et al., 2013; Papadimitriou et al., 2018). In addition, *ACTN3* genotypes were found to have different effects in male and female athletes (Gili et al., 2012). The muscle physiology of female athletes is more affected by ɑ-actinin-3 deficiency than males (Walsh et al., 2008; Zempo et al., 2010). For instance, compared with a general population, female endurance athletes have a greater proportion of XX genotypes, but there was no evidence of this in male endurance athletes (Yang et al., 2003; Shang et al., 2010).

The relationship between *ACTN3* gene and physical performance of football players has received considerable attention from researchers (Sarmento et al., 2020; McAuley et al., 2021). The RR genotype in Brazilian youth and professional players was found to be significantly associated with sprinting speed and explosive power (Pimenta et al., 2013; Coelho et al., 2016; Dionísio et al., 2017). The RR genotype of Italian (Massidda et al., 2012) and Turkish (Atabaş et al., 2020) players was also found to be significantly associated with explosive power. However, Koku et al. (2019) reported that the swing arm vertical jump of European (Caucasians) amateur football players was significantly higher than that of the RR genotype players. This is because the phenotype associated with the same genotype may differ across ethnic groups (Sarmento et al., 2020).

The ability to repeatedly perform high intensity actions has a vital influence on the performance of football players during competition (Dodd and Newans, 2018). In football, players need to perform intermittent high-intensity movements such as sprinting, jumping, turning, kicking and tackling (Stølen et al., 2005). Wide midfielders, fullbacks and forward players are better at sprinting and high-speed movements than central defenders and central midfielders, and wide midfielders cover the longest distances (Tierney et al., 2016; Torreño et al., 2016). Recently, some genetic markers have been found to be associated with football players playing as forwards (Massidda et al., 2018). Clos et al., (2021) reported that the RR genotype and the R allele was the most common in wide midfielders, wingers and forwards. Petr et al. (2022) reported that the *ACTN3* RR genotype in defenders was associated with higher lower limb strength than either the RX or XX genotypes. Previous studies have focused on the association between ACTN3 genotypes and athletic performance in professional players, and the differences in the distribution of *ACTN3* genotypes among players at different positions. However, there have been few studies on the genotype distribution of *ACTN3* in youth football players at different position and its association with athletic performance. Therefore, the aims of this study were: 1), to investigate the prevalence of *ACTN3* polymorphism in elite and sub-elite Chinese football players who played different positions; and, 2) to explore the effects of *ACTN3* polymorphisms on the athletic performance of elite and sub-elite players playing different positions.

## Materials and methods

### Subjects

This study was conducted according to the Declaration of Helsinki and was approved by the Ethics Committee of Fujian Normal University. All subjects, as well as their guardians and football coaches, were made aware of the test process and provided informed consent prior to enrollment in this study.

A total of 166 players were initially recruited from three academic football schools and two campus football clubs in China, and 16 goalkeepers and 3 non-Han players and 5 injured players were excluded. Thus, a total of 74 elite male football players aged 13–15 years from three academic football schools (Jiangsu and Shandong provinces in Eastern China) participating in the National Youth Football Super League, and 68 male football players from two regional football leagues (Jiangsu Province, Eastern China) were ultimately recruited as sub-elite groups. All subjects were of Han Chinese origin. During testing, subjects were permitted to withdraw in the case of injury or simply if they showed unwillingness to undertake field tests.

### Experimental approach

The test was divided into 2 days. On the first morning (7:00–9:00 a.m.), the oral mucosa of the players was collected and stored in a preservation solution. We then measured each player’s height and body mass (on an empty stomach). In the afternoon (15:00–17:00 p.m.), participants sequentially performed 20-m and 30-m sprints, standing long jump, 5 × 25-m repeated sprint, and YYIR1. YYIR1 was conducted after completing the other tests; and the 12-min runs were performed at the same time on the following day.

All subjects recruited had no injuries at the time of the study and they did not take any medicine or inject any drugs during the 30 days prior to this study. They did not consume any alcohol or caffeine during the 48 h before testing. DNA was extracted from the mouth mucosa of every subject and was used to determine the subject’s *ACTN3* R577x genotype by PCR. All raw data for these subjects were collected from September to December 2020.

### Procedures

#### DNA extraction

Mouth mucosa was collected *via* oral flocking swabs (Lang Fu Bio-instrument, Shanghai, China) and placed in a preservation solution. DNA was extracted from mouth mucosa using the TSINGKE silica gel adsorption kit (Qiagen Inc., Valencia, CA, United States).

#### PCR amplification

PCR primers were synthesized according to Malyarchuk et al. (2018). The primers for exon 16 were: forward: 5′-CTG TTG CCT GTG GTA AGT GGG-3′; reverse: 5′-TGG TCA CAG TAT GCA GGA GGG-3′. The components of the amplification system were as follows: Mix (green), 47 uL; 10 μM Primer F, 1 uL; 10 μM Primer R, 1 uL; and Template (gDNA), 1 uL.

The components of the amplification system were as follows: Mix (green), 47 uL; 10 μM Primer F, 1 uL; 10 μM Primer R, 1 uL; and Template (gDNA), 1 uL.

The amplification program consisted of an initial denaturation step at 98°C for 2 min, followed by 30 cycles comprised of 98°C for 10 s, TM°C for 10°s, 72°C for 10°s, and a final extension step at 72°C for 5 min. The amplified PCR products were then subjected to agarose gel electrophoresis (2°uL sample +6°uL bromophenol blue) at 300 V for 12 min.

### Gel extraction of PCR products with DNA gel kit

The reaction system (normal 5 µL) was added in the order of 1°uL primer, 2 µL template, and 1°uL BDT 1 µL betaine. After the completion of inspection, the membrane was covered, and units were balanced in a centrifuge and centrifuged at 4°C and 4000 RPM. We then covered the eight-tube caps, shook and mixed the reaction system, centrifuged the mixture, and placed them in a PCR machine after completion to carry out the reaction. Then, reactions were placed on the PCR instrument for reaction.

The centrifuge plate was added to 38 µL oscillating Ferrite Beads (Tsingke Biotechnology, Guangzhou, Guangdong Province) and rinsed with Magical Buffer (Tsingke Biotechnology, Guangzhou, Guangdong Province). Data from the sequencing machine was then copied and analyze peak data using Sequencing Analysis 5.2.

### Anthropometric measurements

Body sizes were measured with all football players barefoot and wearing pants and a tee shirt. Height was measured with a YL–65S stadiometer (Yagami, Nagoya, Japan) to the nearest 0.1°cm, and body mass was measured with a XiaoMi body-fat monitor (XiaoMi, Beijing, China) to the nearest 0.1 kg.

### Sprint measurements

Sprint times were assessed over 20-m and 30-m using timing gates (Brower Timing System, Draper, UT, United States). Subjects started 0.5 m behind the start line and ran maximally past the 30-m timing gate. Times were recorded to the nearest 0.01 s with the quicker of two attempts being used for the sprint score. The intra-rater reliability ICCs (3, 1) for the 20-m sprint and 30-m sprint tests were 0.9 and 0.89, respectively.

### Jump measurement

Standing long jump was carried out to assess the players’ explosive muscular power. All football players placed both feet behind a starting line and jumped as far as possible, while landing on both feet. The distance from the line to the player’s closest heel was then measured with a measuring tape. The test was performed twice, and the longest jump distance between the two measurements was used for analysis. The intra-rater reliability ICC (3, 1) for the standing long jump tests was 0.86.

### Speed endurance measurement

A 5 × 25-m repeated sprint was used to evaluate anaerobic endurance. On a 25-m straight line, marker barrels were placed at intervals of 5 m. On hearing the command to “run”, the participants ran from the starting line to the first marker barrel and knocked it down with their hands, then turned back to the starting line and again knocked down the marker barrel with their hands. This task was repeated for the second and remaining marker barrels. The runners sequentially knocked down all the marker barrels, and ultimately sprinted back to the starting line. Times were recorded to the nearest 0.01 s.

### Football-specific endurance measurements

Krustrup et al. (2003) reported YoYo intermittent recovery level 1 (YYIR1) to be a valid and reliable test in assessing specific fitness for football. During the test, the players performed a series of 20-m runs—resting for 5 s every 40 m—and then ran with progressive increments in speed and for increasingly shorter time periods between changes. Failure to achieve the shuttle run in time on two occasions resulted in termination of the test. We therefore recorded the distance when the players failed to cross the finish line on time for the second time.

### Aerobic fitness measurements

The Cooper’s 12 min running test is a popular field test used to measure aerobic fitness, and it is also used to estimate an athlete’s VO_2_ max (Conley et al., 1991). Here, the subjects were required to run as many laps as possible on a 400-m track for 12 min. After 12 min, the subjects were asked to stop running. Then, we recorded the subject’s total distance (in meters) covered after 12 min. VO_2_ max was predicted using the following formula:

VO_2_ max (mL/kg/min) = (22.351 x distance covered in kilometers) - 11.288.

### Statistical analyses

Chi-square (χ^2^) tests were employed to test for Hardy-Weinberg equilibrium (HWE). χ^2^ tests were also used to test both the allele frequencies between the two groups under the significance level at *p* ≤ 0.05. Fisher’s exact test was used to determine whether there were differences in the distribution of *ACTN3* R and X alleles between elite and sub-elite football players. One-way analysis of variance was used to analyze the different physical performance between the RR, RX, and XX groups, and Bonferroni’s *post hoc* test was performed to determine which measurements were significantly different. Independent samples *t*-tests were used to examine differences in anthropometrics and physical indicators between elite and sub-elite players. All statistical analyses were conducted using IBM-SPSS 26.0 for Windows.

## Results

### Population

The 142 players were divided into defenders, midfielders and forwards. Elite defenders, midfielders and forward players were found to be taller (*p* < 0.001, *p* = 0.022, *p* = 0.019, respectively) and heavier (*p* < 0.001, *p* = 0.014, *p* = 0.023, respectively) than sub-elite players (Table 1).

**TABLE 1:** Anthropometric characteristics of all subjects in different positions.

Positions	Overall			Defender			Midfielder			Forward player		
Groups	Elite (n = 74)	Sub-elite (n = 68)	*p*	Elite (n = 32)	Sub-elite (n = 24)	*p*	Elite (n = 24)	Sub-elite (n = 21)	*p*	Elite (n = 18)	Sub-elite (n = 23)	*p*
Age	13.89 ± 0.79	13.66 ± 0.78	0.083	13.91 ± 0.86	13.71 ± 0.81	0.384	13.88 ± 0.68	13.67 ± 0.86	0.368	13.89 ± 0.83	13.61 ± 0.72	0.256
Heigh (cm)	172.7 ± 7.53	167.0 ± 9.02	<0.001	174.0 ± 6.86	168.8 ± 9.73	0.022	171.7 ± 6.72	167.0 ± 6.04	0.019	171.9 ± 9.54	165.02 ± 10.38	0.035
Body mass (kg)	57.81 ± 8.70	51.18 ± 8.10	<0.001	58.69 ± 7.65	52.73 ± 9.97	0.014	55.56 ± 7.95	50.63 ± 5.69	0.023	59.27 ± 11.03	50.07 ± 7.88	0.003

### Prevalence of the R577x polymorphism among players at different positions

The genotype distribution of the R577X polymorphism in both 74 elite (χ^2^ = 3.024; df = 1; *p* = 0.082) and 68 sub-elite football players (χ^2^ = 0.979; df = 1; *p* = 0.322) were in accordance with Hardy-Weinberg equilibrium based on Chi-square (χ^2^) tests, which showed that the two groups of subjects selected in this experiment had the characteristics of group representation (*p* > 0.05).

Tables 2, 3 summarizes the distribution of *ACTN3* genotypes and allele frequencies in elite and sub-elite players at different positions. Genotype RX (39.4%) was the most common among all players. Genotype RR (44.6%) was the most common in elite players, and RX (42.6%) was the most common in sub-elite players. There were significant differences in RR, RX and XX genotypes between elite and sub-elite players (χ^2^ = 12.135, df = 2, *p* = 0.02). There was a significant difference in the RR genotype distribution between elite and sub-elite players (χ^2^ = 8.696, df = 1, *p* = 0.03). There were also significant differences in RR, RX, and XX genotypes between elite and sub-elite midfielders (χ^2^ = 9.129, df = 2, *p* = 0.01). Moreover, there were significant differences in the RR genotype distributions between elite and sub-elite midfielders and defenders (χ^2^ = 8.696, df = 1, *p* = 0.03; χ^2^ = 5.556, df = 1, *p* = 0.018, respectively).

**TABLE 2:** The comparison of genotype frequencies in each elite and sub-elite goalkeeper, defender, midfielders and forward player.

Groups	Positions		RR	RX	XX	Overall	χ^2^	df	*p*
Overall	Defender	n	18	20	18	53	0.143	2	0.931
		%	32.1	35.7	32.1	100			
	Midfielder	n	14	18	13	45	0.933	2	0.627
		%	31.1	40.0	28.9	100			
	Forward	n	14	18	9	41	2.976	2	0.226
		%	34.1	43.9	22.0	100			
	Overall	n	46	56	40	142	2.761	2	0.252
		%	32.4	39.4	28.2	100			
Elite	Defender	n	14	11	7	32	2.313	2	0.315
		%	43.8	34.4	21.9	100			
	Midfielder	n	12	8	4	24	4.0	2	0.135
		%	50.0	33.3	16.7	100			
	Forward	n	7	8	3	18	2.333	2	0.311
		%	38.9	44.4	16.7	100			
	Overall	n	33	27	14	74	7.649	2	0.022
		%	44.6	36.5	18.9	100			
Sub-elite	Defender	n	4	9	11	24	3.250	2	0.197
		%	16.7	37.5	45.8	100.0			
	Midfielder	n	2	10	9	21	5.429	2	0.066
		%	9.5	47.6	42.9	100			
	Forward	n	7	10	6	23	1.130	2	0.568
		%	30.4	43.5	26.1	100			
	Overall	n	13	29	26	68	6.382	2	0.041
		%	19.1	42.6	38.2	100			

**TABLE 3:** The comparison of allele frequencies in each elite and sub-elite goalkeeper, defender, midfielders and forward player.

Groups	Positions		R	X	χ^2^	df	*p*
Overall	Defender	N	56	56	0.0	1	1.0
		%	50	50			
	Midfielder	N	46	44	0.044	1	0.833
		%	51.1	48.9			
	Forward	N	46	36	1.22	1	0.269
		%	56.1	43.9			
	Overall	N	148	136	0.507	1	0.476
		%	52.1	47.9			
Elite	Defender	N	39	25	3.063	1	0.08
		%	60.9	39.1			
	Midfielder	N	32	16	5.333	1	0.021
		%	66.7	33.3			
	Forward	N	22	14	1.778	1	0.182
		%	61.1	38.9			
	Overall	N	93	55	9.757	1	0.002
		%	62.8	37.2			
Sub-elite	Defender	N	17	31	4.083	1	0.043
		%	35.4	64.6			
	Midfielder	N	14	28	4.667	1	0.031
		%	33.3	66.7			
	Forward	N	24	22	0.087	1	0.768
		%	52.2	47.8			
	Overall	N	55	81	4.971	1	0.026
		%	40.4	59.6			

There were significant differences in R and X alleles (χ^2^ = 14.246, df = 1, *p* < 0.001) between elite and sub-elite players. There were significant differences in R and X alleles between elite and sub-elite defenders (χ^2^ = 7.146, df = 1, *p* = 0.008) and midfielders (χ^2^ = 9.96, df = 1, *p* = 0.002). These results indicated that the RR genotypes and R alleles were highly represented among elite defenders and midfielders.

### Physical performance in players with different genotypes at different positions

As shown in Table 4, in all subjects, RR players performed better 5 × 25-m repeat sprint ability (*p* = 0.064, *p* = 0.007), in standing long jump (*p* = 0.005; *p* = 0.02), 20-m sprint (*p* = 0.001; *p* = 0.034) and 30-m sprint (*p* < 0.001; *p* = 0.06) than RX or XX players, and RR players also performed better than X allele players 5 × 25-m repeat sprint ability (*p* = 0.014), in standing long jump (*p* = 0.003), 20-m sprint (*p* = 0.001) and 30-m sprint (*p* < 0.001). There was no significant difference in YYIR1 or VO_2_ max between RR, RX and XX players.

**TABLE 4:** Comparison of athletic performances of RR, RX and XX genotype in elite and sub-elite goalkeeper, defender, midfielder and forward players.

Index	Groups	RR	RX	XX	*p*	RX + XX	*p* (RRvs RX + XX)
5 × 25-m RSA (s)	Overall	35.03 ± 1.81^b^	35.86 ± 2.44	36.04 ± 1.51^b^	0.044	35.93 ± 2.10	0.014
	Elite	34.53 ± 1.66	34.78 ± 2.07	35.54 ± 1.50	0.215	35.04 ± 1.91	0.218
	Sub-elite	36.32 ± 1.57	36.84 ± 2.37	36.32 ± 1.47	0.545	36.59 ± 1.99	0.649
SLJ (cm)	Overall	225.3 ± 17.85^a^	214.8 ± 18.37^a^	216.1 ± 18.34	0.010	215.3 ± 18.27	0.003
	Elite	228.9 ± 15.29	225.0 ± 16.95	232.1 ± 16.45	0.379	227.4 ± 16.92	0.694
	Sub-elite	216.1 ± 21.05	205.4 ± 14.24	207.4 ± 12.69	0.109	206.3 ± 13.45	0.04
20 m	Overall	3.25 ± 0.24^a^ ^,^ ^b^	3.43 ± 0.28^a^	3.36 ± 0.24^b^	0.002	3.40 ± 0.26	0.001
	Elite	3.22 ± 0.25	3.29 ± 0.24	3.21 ± 0.19	0.478	3.26 ± 0.23	0.459
	Sub-elite	3.31 ± 0.21^a^	3.55 ± 0.25^a^	3.44 ± 0.22	0.008	3.50 ± 0.24	0.013
30 m	Overall	4.57 ± 0.30^a^ ^,^ ^b^	4.82 ± 0.40^a^	4.77 ± 0.37^b^	0.002	4.80 ± 0.39	<0.001
	Elite	4.52 ± 0.30	4.60 ± 0.29	4.54 ± 0.22	0.507	4.58 ± 0.27	0.367
	Sub-elite	4.68 ± 0.28^a^	5.03 ± 0.39^a^	4.90 ± 0.38	0.021	4.97 ± 0.38	0.014
YYIR1	Overall	1830.9 ± 325.9	1716.3 ± 364.7	1806.5 ± 294.1	0.190	1753.9 ± 338.4	0.201
	Elite	1878.8 ± 308.4	1728.2 ± 387.8	1924.3 ± 290.5	0.127	1795.1 ± 366.1	0.299
	Sub-elite	1709.2 ± 349.9	1705.2 ± 348.5	1743.1 ± 281.2	0.902	1723.1 ± 316.1	0.890
VO2max	Overall	54.7 ± 4.59	54.26 ± 4.21	55.02 ± 3.62	0.677	54.58 ± 3.98	0.871
	Elite	55.16 ± 5.19	55.22 ± 2.93	56.16 ± 3.58	0.740	55.54 ± 3.16	0.703
	Sub-elite	53.52 ± 2.25	53.37 ± 5.02	54.40 ± 3.56	0.625	53.86 ± 4.38	0.786

Notes: SLJ, standing long jump; 5 × 25-m RSA, 5 × 25-m repeated sprint ability; YYIR1, YoYo intermittent recovery test level 1.

^a^ Significant difference between the RR, and RX, genotype groups: *p* < 0.05.

^b^ Significant difference between the RR, and XXgenotype groups: *p* < 0.05.

There was no significant difference in the 5 × 25-m repeat sprint ability, VO_2_ max, standing long jump, 20-m sprint, 30-m sprint and YYIR1 between RR, RX and XX elite players. For the RR sub-elite players, the 20-m (*p* = 0.004) and 30 m (*p* = 0.006) sprint times were significantly shorter than RX players (Table 4).

As shown in Table 5, elite players had faster 20-m (*p* = 0.002)/30-m sprints (*p* = 0.003) and 5 × 25-m RSA (*p* < 0.001), longer standing long jumps (*p* < 0.001), longer YYIR1 distances (*p* = 0.046) and higher VO2 max than sub-elite players (*p* = 0.024). The RR, RX and XX elite players’ standing long jumps were farther (*p* = 0.026, *p* < 0.001 and *p* < 0.001, respectively) than sub-elite players. The 5 × 25-m RSA time of RR and RX elite players was significantly shorter (*p* = 0.02, *p* = 0.001, respectively) than that of sub-elite players. In addition, the 20-m (*p* < 0.001, *p* = 0.003) and 30-m sprint times (*p* < 0.001, *p* = 0.002) of RX and XX elite players were significantly less than that of sub-elite players. There was no significant difference in the VO_2_ max and YYIR1 between RR, RX, and XX elite and sub-elite players.

**TABLE 5:** Comparison of RR, RX and XX of athletic performance in elite and sub-elite players.

	Genotypes	Groups	5 × 25 m RSA (s)	SLJ (m)	20 m (s)	30 m (s)	YYIR1 (m)	VO_2_ max (mL/kg/min)
Overall	Overall	Elite	34.80 ± 1.81	228.1 ± 16.13	3.24 ± 0.24	4.55 ± 0.28	1832.4 ± 39.7	55.37 ± 4.16
		Sub-elite	36.54 ± 1.91	208.2 ± 15.49	3.46 ± 0.25	4.91 ± 0.38	1720.4 ± 320.2	53.79 ± 4.05
		*p*	<0.001	<0.001	0.002	0.003	0.046	0.024
	RR	Elite	34.53 ± 1.66	228.9 ± 15.29	3.22 ± 0.25	4.52 ± 0.30	1878.8 ± 308.4	55.16 ± 5.19
		Sub-elite	36.32 ± 1.57	216.08 ± 21.05	3.31 ± 0.21	4.68 ± 0.28	1709.2 ± 349.9	53.52 ± 2.25
		*P*	0.002	0.026	0.256	0.106	0.113	0.278
	RX	Elite	34.78 ± 2.07	225.0 ± 16.95	3.29 ± 0.24	4.60 ± 0.29	1728.1 ± 387.5	55.22 ± 2.93
		Sub-elite	36.84 ± 2.37	205.3 ± 14.24	3.55 ± 0.25	5.03 ± 0.39	1705.2 ± 348.5	53.37 ± 5.02
		*P*	0.001	<0.001	<0.001	<0.001	0.816	0.102
	XX	Elite	35.53 ± 1.50	232.1 ± 16.45	3.21 ± 0.19	4.54 ± 0.22	1924.3 ± 290.5	56.16 ± 3.58
		Sub-elite	36.32 ± 1.47	207.4 ± 12.69	3.44 ± 0.22	4.90 ± 0.38	1743.1 ± 281.2	54.40 ± 3.56
		*p*	0.12	<0.001	0.003	0.002	0.062	0.146
Defenders	RR	Elite	34.24 ± 1.71	229.6 ± 14.29	3.21 ± 0.32	4.51 ± 0.40	1818.6 ± 312.75	55.21 ± 4.89
		Sub-elite	37.0 ± 1.08	225.3 ± 28.69	3.28 ± 0.13	4.57 ± 0.16	1710.0 ± 471.5	54.70 ± 3.45
		*P*	0.008	0.671	0.695	0.760	0.590	0.850
	RX	Elite	34.18 ± 2.02	226.5 ± 18.68	3.28 ± 0.27	4.54 ± 0.28	1861.8 ± 294.4	54.93 ± 3.48
		Sub-elite	37.05 ± 3.77	208.1 ± 16.26	3.55 ± 0.29	4.98 ± 0.35	1737.8 ± 336.7	49.68 ± 6.97
		*P*	0.043	0.032	0.044	0.006	0.391	0.042
	XX	Elite	35.13 ± 1.37	222.7 ± 14.56	3.28 ± 0.15	4.61 ± 0.15	2062.9 ± 161.4	56.86 ± 3.82
		Sub-elite	36.68 ± 1.15	211.5 ± 15.25	3.43 ± 0.14	4.95 ± 0.42	1747.3 ± 279.6	53.68 ± 3.40
		*P*	0.019	0.143	0.044	0.058	0.016	0.083
Midfielders	RR	Elite	35.03 ± 1.92	229.3 ± 14.67	3.20 ± 0.20	4.48 ± 0.23	1900.0 ± 357.3	54.06 ± 6.30
		Sub-elite	33.72 ± 1.96	208.5 ± 16.26	3.35 ± 0.54	4.75 ± 0.64	1430.0 ± 325.3	54.09 ± 2.38
		*P*	0.405	0.092	0.432	0.243	0.108	0.996
	RX	Elite	34.29 ± 2.02	224.3 ± 13.83	3.18 ± 0.23	4.53 ± 0.32	1530.0 ± 433.3	54.96 ± 1.70
		Sub-elite	36.56 ± 1.80	204.5 ± 10.92	3.51 ± 0.20	5.04 ± 0.45	1698.0 ± 342.6	54.90 ± 3.35
		*P*	0.023	0.002	0.005	0.016	0.371	0.960
	XX	Elite	36.27 ± 1.72	234.8 ± 9.5	3.14 ± 0.22	4.47 ± 0.31	1825.0 ± 216.3	55.72 ± 4.84
		Sub-elite	36.35 ± 1.69	206.0 ± 9.15	3.41 ± 0.21	4.83 ± 0.28	1693.3 ± 353.1	55.72 ± 4.35
		*P*	0.933	<0.001	0.073	0.065	0.510	0.999
Forward players	RR	Elite	34.25 ± 0.86	227.0 ± 20.12	3.28 ± 0.16	4.62 ± 0.19	1962.9 ± 208.9	59.96 ± 3.62
		Sub-elite	36.68 ± 0.74	213.0 ± 18.55	3.32 ± 0.18	4.72 ± 0.25	1788.6 ± 287.7	52.67 ± 1.16
		*P*	<0.001	0.201	0.693	0.378	0.219	0.011
	RX	Elite	36.08 ± 1.79	223.6 ± 19.26	3.41 ± 0.19	4.76 ± 0.23	1742.5 ± 416.7	55.87 ± 3.33
		Sub-elite	36.94 ± 1.20	203.7 ± 16.29	3.61 ± 0.27	5.02 ± 0.41	1683.0 ± 397.4	55.17 ± 2.03
		*P*	0.239	0.03	0.102	0.081	0.761	0.587
	XX	Elite	35.53 ± 1.72	250.7 ± 12.22	3.16 ± 0.25	4.45 ± 0.25	1733.3 ± 508.5	55.10 ± 0.81
		Sub-elite	35.60 ± 1.64	201.83 ± 11.30	3.48 ± 0.35	4.91 ± 0.47	1810.0 ± 172.4	53.75 ± 2.28
		*p*	0.955	0.001	0.200	0.161	0.735	0.367

RR, RX, and XX elite defenders’ 5 × 25 m RSA times were all faster than those of sub-elite players, and RX elite defenders’ standing long jumps were longer (*p* = 0.032), as well as their 20 m (*p* = 0.044) and 30 m (*p* = 0.006) sprints being faster.

RX elite midfielders’ 5 × 25-m RSA and 20-m and 30-m sprint times were faster than sub-elite players (*p* = 0.023, *p* = 0.044, *p* = 0.006, respectively). RX and XX elite midfielders had longer standing jumps than sub-elite players (*p* = 0.002, *p* < 0.001, respectively).

RR elite forward players’ 5 × 25-m RSA times were faster than those of sub-elite players (*p* < 0.001), and RX and XX players had longer standing long jumps than sub-elite players (*p* = 0.003, *p* = 0.001).

## Discussion

This is the first study to explore the genotype and allele distribution of youth football players with different competitive statuses and field positions. The *ACTN3* R577x polymorphism has been the most consistent gene that has been shown to affect athletic performance, especially at the elite football players (McAuley et al., 2021). Our results show that the frequency of elite players in the RR genotype and R allele were significantly higher than that of sub-elite players. This is similar to the distribution of *ACTN3* genotype and allele distribution of the Brazilian youth football players (Coelho et al., 2018). The RR genotype and R allele distribution frequency of elite defenders and midfielders were significantly higher than those of sub-elite players.

For all subjects in the present study, the heterozygous genotype RX was the most common, consistent with previous studies involving professional players (Massidda et al., 2014; Dionísio et al., 2017; Coelho et al., 2018; Clos et al., 2021). We also found the RR genotype to be more prevalent than RX and XX genotype, and RR players performed better in 20-m/30-m sprints, the 5 × 25 = *m* RSA and the standing long jump. There was no significant in football-specific endurance or VO_2_ max between RR, RX and XX players. Considering that a player’s strength/power and speed have a crucial effect on the outcome of a game (Stølen et al., 2005), and given the higher physical demands of training and games in high-level competition, the higher frequency of the RR genotype in this group of elite players is not surprising (McAuley et al., 2021). Our results were consistent with a previous study on elite football players (Santiago et al., 2008; Coelho et al., 2018; Massidda et al., 2019).

These results suggest that the RR genotype is correlated with football players who display both superior speed and explosive muscle power, which is consistent with previous studies on football players (Zois et al., 2011; Ahmetov et al., 2014; Honarpour et al., 2017). Although there have been few studies on the mechanism of *ACTN3* polymorphisms with regards to the physiology of human muscles, an Actn3^−/−^ knockout mouse model has been utilized to explain the mechanism by which *α*-actin-3 alters skeletal muscle function (MacArthur et al., 2007). Compared with wild type mice, *ACTN3* knockout mice exhibited decreased fast-twitch fiber diameter and decreased muscle mass. Moreover, their grip strength was significantly reduced and their endurance was significantly diminished (MacArthur et al., 2008). Anatomical analysis revealed that the activities of anaerobic metabolic enzymes decreased and the activities of aerobic metabolic enzymes increased in the fast-twitch muscle fibers of knockout mice, while the distribution of muscle fiber types did not change significantly (MacArthur et al., 2008). Vincent et al. (2007) reported that the proportion of cross-sectional area and the number of type IIx muscle fibers (fast twitch) in males with the RR genotype was significantly greater than those from XX genotypes, suggesting that the RR genotype was beneficial to the formation of fast-twitch muscle fibers. Ahmetov et al. (2014) reported that the testosterone level of RR genotype athletes in static states was significantly greater than that of players with the XX genotype. Therefore, RR genotype athletes possess greater explosive power and have a higher probability to become elite athletes, especially in sporting events that require the combination of both endurance and strength (Ahmetov et al., 2014).

This study found that there was no significant difference in the physical performance between RR, RX and XX elite players, while the 20-m and 30-m sprint times of RR sub-elite players were significantly shorter than those of RX sub-elite players. This result suggests that the RR genotype has a significant effect on the physical abilities of Chinese football players (except YYIR1 and VO_2_ max), but it had no effect on elite players. However, Yang et al. (2017) studied Chinese elite athletes and suggested that the RR genotype was widely distributed in Chinese international-level spring/power athletes, while athletes with the RR genotype had superior lower-extremity power than either those with the RX or XX genotypes. Training experience may also be an important factor for elite or sub-elite athletes (Heffernan et al., 2016). For example, there has been no association shown between the *ACTN3* gene and power and strength test results in elite volleyball (Ruiz et al., 2011) and basketball players (Garatachea et al., 2014). This may be due to the fact that volleyball and basketball players must be able to jump high, so they focus on developing this specific ability in their training (Heffernan et al., 2016).

Previous studies have shown that the R allele of *ACTN3* R577X was the dominant allele for athletes with strength and speed (Yang et al., 2003; Roth et al., 2008). Moreover, athletes with the R allele have higher testosterone levels at rest (Ahmetov et al., 2014). Therefore, the athletes with the R allele are more likely to become power-related elite athletes. Using genome-wide association study (GWAS) analysis, Chen et al. (2009) concluded that the one-dimensional, north-south structure of the Chinese Han population is characterized by a continuous gradient, rather than by distinct subpopulation groups. All subjects in the current study were from the Han Chinese population found in Eastern China, and the genotype and allele distribution of ACTN3 would, therefore, not be affected by ethnic or regional differences. This investigation demonstrated the existence of distinguished differences in the RR genotype (χ^2^ = 8.696, df = 1, *p* = 0.03) and R allele (χ^2^ = 14.246, df = 1, *p* < 0.001) frequency between elite and sub-elite football players. The RR genotype (44.6% vs. 18.8%) and R allele (62.8% vs. 37.2%) frequency in elite football players was significantly higher than the X allele frequency. Therefore, this current study provides evidence that the RR genotype and R allele are the dominant allele in Chinese elite football players. The findings of this study were similar to the results from Wei (2021) who studied the distribution of *ACTN3* genotypes in Chinese elite female soccer players, although the R and X alleles of Chinese elite female footballers were not significantly different from control subjects (non-athlete Chinese female). This may be related to the different levels of competition in the control subjects.

Enhancing the ability to perform repetitive high intensity actions is a vital part to improving the athletic ability of football players. Football players need to repeat sprints (Girard and Mendez-Villanueva, 2011), jumps and other actions that require high-intensity muscle work, and also need to have endurance capabilities (Berman and North, 2010; Eynon et al., 2014). Assuming that the R allele was associated with strength and speed, the X allele may be associated with the athletic ability of endurance (Druzhevskaya et al., 2008). Furthermore, it can be inferred that RX genotype was also a dominant genotype in elite football players. Previous studies have also supported this assumption (Dionísio et al., 2017; Coelho et al., 2018; Clos et al., 2021). Despite this, there have been few studies on the influence of RX genotype on athletic ability. This study found that there was no significant difference in the RX genotype between elite and sub-elite football players at different positions.

Training experience may be an important factor for being an elite or sub-elite athlete (Heffernan et al., 2016). For example, no association has been shown between the *ACTN3* gene and power and strength test results in elite volleyball (Ruiz et al., 2011) and basketball players with XX genotype, which was found to be the dominant genotype influencing muscle endurance (Yang et al., 2003; Niemi and Majamaa, 2005; Massidda et al., 2015). However, the present study suggests that RR players have better physical performance than RX or XX players in many measures other than YYIR1 and VO_2_ max. In addition, RR elite players performed better at 5 × 25-m RSA and standing long jump, and RX and XX elite players performed better at standing long jump and 20-m/30-m sprints than sub-elite players. Therefore, we posit that not all athletes having a dominant genotype or alleles are likely to become elite football players.

Some studies have also reported that *ACTN3* R577x is not associated with team sports such as football (Santiago et al., 2008; Coelho et al., 2018). The success of football players in their football career is also influenced by many other factors such as football technique, tactical consciousness and psychological skills. Thus, it remains necessary to use caution when using genes to predict the future development of football players or to identify gifted football players at early ages. Even if a genetic profile is the most important factor that influences the successful career of football players, there are other factors that should be involved in any selection mode (Rees et al., 2016).

Based on the above, this study found that the RR genotype frequency of elite players was significantly higher than that of sub-elite players. In particular, the RR genotype distribution frequency of elite defenders and midfielders was significantly higher than that of sub-elite. RR genotype players were significantly better than RX or XX players in terms of speed and explosive power. Therefore, this study suggests that the RR genotype plays an important complementary role in the identification of physical talent in elite Chinese football players. In addition, we posit that a given player’s training plan and recovery period can be adjusted throughout the season based on their *ACTN3* genotype (Maffulli et al., 2013; Baumert et al., 2016; Jones et al., 2016). This information can also reduce the probability of sports injuries to players. For example, it has been accepted that the XX genotype in *ACTN3* individuals including football players have a higher susceptibility to muscle micro-trauma after performing some eccentric exercises (Lek and North, 2010; Massidda et al., 2019). The results of this study can provide football schools with a tool for the identification of physical talent in youth players and guide the improvement of training in youth players who are already at the elite level.

However, studies in recent years have shown that individual *ACTN3* gene polymorphisms did not contribute significantly to physical performance (Moran et al., 2007; Gineviciene et al., 2016). Thus, the combination of two or more genes may be better associated with the relationship between genes and physical performance. Erskine et al. (2014) demonstrated that males with the “optimal” *ACE/ACTN3* genotype combination (*ACTN3* RR or RX + *ACE* DD or ID) had greater maximum isoinertial strength (1—RM) and maximum power than those with a “sub-optimal” profile. The combination of *ACE* and *ACTN3* genotypes (*ACE* II/ID/DD + *ACTN3* RR/XR) in Chinese elite female football players also had significantly better VO_2_ max than a control group (Wei, 2021). Therefore, there is a need to investigate the effects of other genes and gene combinations on the athletic performance of Chinese football players in the future.

The present study has some limitations. First, the sample size is small. The smaller sample size may affect the accuracy of the results, and the value of the significant differences may also decrease (Bouchard, 2011). Second, the elite players were from three different teams, and the equipment used for physical training, the practice environment, and coaching styles may have varied considerably between teams. These factors also play an important role when assessing the association between genotype and athletic performance. In addition, 13–15-year-old players are undergoing a period of rapid growth, which affects both their anthropometric and athletic performance, both of which will affect the players’ most suitable position in the future. Moreover, some players play several positions according to tactical needs.

## Conclusion

We here identified for the first time an association between *ACTN3* R577x polymorphism and defenders and midfielders in Chinese youth football players. These findings need to be replicated in other cohorts of elite football players with different ethnic and geographic backgrounds. In addition, this study holds that the RR genotype is correlated with the speed and explosive power of Chinese youth football players. Given that youth football players are still growing and developing, this study may help coaches understand the genes influencing football players and positioning. However, we should take into account that football is a very complex multifactorial trait influenced by many genes, environmental factors, and the actions. The *ACTN3* polymorphism is one of the many genetic variants influencing the athletic performance of football players.

## Data Availability

The original contributions presented in the study are included in the article/Supplementary Material, further inquiries can be directed to the corresponding author.
